# Glycerol-3-Phosphate Acyltransferase 3 (OsGPAT3) is required for anther development and male fertility in rice

**DOI:** 10.1093/jxb/erw445

**Published:** 2017-01-12

**Authors:** Xiao Men, Jianxin Shi, Wanqi Liang, Qianfei Zhang, Gaibin Lian, Sheng Quan, Lu Zhu, Zhijing Luo, Mingjiao Chen, Dabing Zhang

**Affiliations:** 1 State Key Laboratory of Hybrid Rice, Shanghai Jiao Tong University and University of Adelaide Joint Centre for Agriculture and Health, School of Life Sciences and Biotechnology, Shanghai Jiao Tong University, Shanghai, China; 2 School of Agriculture, Food and Wine, University of Adelaide, Urrbrae, South Australia, Australia

**Keywords:** Anther development, glycerol-3-phosphate acyltransferase, lipid metabolism, male sterility, microgametophyte, rice, tapetum

## Abstract

Lipid molecules are key structural components of plant male reproductive organs, such as the anther and pollen. Although advances have been made in the understanding of acyl lipids in plant reproduction, the metabolic pathways of other lipid compounds, particularly glycerolipids, are not fully understood. Here we report that an endoplasmic reticulum-localized enzyme, Glycerol-3-Phosphate Acyltransferase 3 (OsGPAT3), plays an indispensable role in anther development and pollen formation in rice. *OsGPAT3* is preferentially expressed in the tapetum and microspores of the anther. Compared with wild-type plants, the *osgpat3* mutant displays smaller, pale yellow anthers with defective anther cuticle, degenerated pollen with defective exine, and abnormal tapetum development and degeneration. Anthers of the *osgpat3* mutant have dramatic reductions of all aliphatic lipid contents. The defective cuticle and pollen phenotype coincide well with the down-regulation of sets of genes involved in lipid metabolism and regulation of anther development. Taking these findings together, this work reveals the indispensable role of a monocot-specific glycerol-3-phosphate acyltransferase in male reproduction in rice.

## Teaser

Dysfunction of OsGPAT3 leads to disrupted anther lipid metabolism and male sterility in rice.

## Introduction

Male reproductive development in higher plants is a complicated biological process that includes the development of the anther and the generation of pollen (Liu and Qu, 2008; Ma, 2005; Sanders *et al.*, 1999; Zhang *et al.*, 2011; Zhang and Wilson, 2009). The developed anther wall has four somatic layers: the epidermis, the endothecium, the middle layer, and the tapetum (Goldberg *et al.*, 1993). The innermost cell layer of the anther wall, the tapetum, which encompasses the meiotic cells (microsporocytes) at the center, plays a crucial role in regulating programmed anther development and microspore/pollen formation (Li *et al.*, 2006; Parish and Li, 2010; Zhang and Yang, 2014). Tapetal cell differentiation and tapetum development are critical for the early events in male reproduction, including meiosis, while tapetal degeneration is vital for formation of viable pollen during late pollen development (Ma, 2005; Wilson and Zhang, 2009; Zhang and Liang, 2016; Zhang *et al.*, 2011). Tapetal cells are characterized by the presence of abundant organelles and vigorous metabolic activities, secreting various monomers or precursors for the synthesis of the anther cuticle, pollen wall, and pollen intracellular nutrients (Li *et al.*, 2010; Liu and Fan, 2013; Zhang and Li, 2014; Zhang *et al.*, 2011; Zhang and Yang, 2014). The disruption of tapetal function frequently leads to aborted microspore formation and male sterility (Fu *et al.*, 2014; Gómez *et al.*, 2015; Li *et al.*, 2006; Li *et al.*, 2011; Niu *et al.*, 2013; Parish and Li, 2010; Xu *et al.*, 2010; Zhang *et al.*, 2008; Zhang *et al.*, 2010). On the other hand, the mature pollen that is produced within the anther is covered by a specialized pollen wall comprised mainly of three layers: the pollen coat, exine, and intine. Pollen serves as a biological protector of male sperm cells and a communicator between male and female reproductive organs that facilitates pollination and fertilization. The disruption of pollen formation often results in male sterility (Dobritsa *et al.*, 2009; Shi *et al.*, 2015). Recent studies have demonstrated that there is a high level of conservation between anther development and pollen formation in plants, highlighting their joint function in plant reproduction (Gómez *et al.*, 2015; Huang *et al.*, 2015).

Two lipid layers play important protective roles in plant anther development and pollen formation. The first of these is the anther epidermal cuticle and the second is the pollen exine. Both layers share a common lipid metabolic pathway similar to those found in plant cuticular lipids, waxes, and cutin (or suberin) (Jeffree, 1996; Kunst and Samuels, 2003; Li and Zhang, 2010; Li-Beisson *et al.*, 2013; Nawrath, 2006; Zhang *et al.*, 2016), which seems to be conserved at least in land plants (Shi *et al.*, 2015; Wallace *et al.*, 2015). Therefore, cuticular lipid metabolism, including biosynthesis, modification, transport, and transcription regulation, is essential for the normal development of anther and microspores (Shi *et al.*, 2015; Zhang and Li, 2014).

During the past decades, significant advances in the understanding of lipid metabolism in plant reproductive development and male fertility have been achieved on the basis of studies of mutants of various lipid metabolic genes, mainly in Arabidopsis and rice; for example, *Male Sterile 2* (*MS2*) (Aarts *et al.*, 1997; Chen *et al.*, 2011*a*), *CYP703A2* (Morant *et al.*, 2007), *CYP704B1* (Dobritsa *et al.*, 2009), *ACOS5* (de Azevedo Souza *et al.*, 2009), and *WBC2*7 (Dou *et al.*, 2011) in Arabidopsis, and *Defective Pollen Wall* (*DPW*) (Shi *et al.*, 2011), *CYP703A3* (Aya *et al.*, 2009; Yang *et al.*, 2014), *CYP704B2* (Li *et al.*, 2010), *Wax-deficient anther1* (*WDA1*) (Jung *et al.*, 2006), *OsC6* (Zhang *et al.*, 2010), *PDA1*/*OsABCG15* (Qin *et al.*, 2013; Wu *et al.*, 2014; Zhu *et al.*, 2013), *DPW2* (Xu *et al.*, 2016), *OsDEX1* (Yu *et al.*, 2016), and *OsABCG26* (Zhao *et al.*, 2015) in rice. Except *WDA1*, most of these genes have relatively high expression levels in the tapetum and/or microspores, and their corresponding mutants exhibit defective anther cuticle and/or pollen exine together with the reduction of anther cuticular lipid components and pollen wall lipid constituents, causing complete or partial male sterility (see review by Shi *et al.*, 2015). Transcription factors that govern tapetum development in plants also play important roles in regulating cuticular lipid-mediated anther and pollen development, such as *ABORTED MICROSPORES* (*AMS*) (Xu *et al.*, 2014; Xu *et al.*, 2010) in Arabidopsis and *Tapetum Degeneration Retardation* (*TDR*) in rice (Li *et al.*, 2006; Zhang *et al.*, 2008). Nevertheless, none of the abovementioned genes and transcription factors is involved in the metabolism of glycerolipids, an important cutin component in plants (Graça *et al.*, 2002; Li-Beisson *et al.*, 2013), let alone the genetic, biochemical, and molecular mechanisms underlying the involvement of glycerolipid metabolism in anther and pollen development.

The glycerolipid triacylglycerol (TAG) and its derivatives are important storage and membrane lipids and indispensable components of biological polymers including cutin and suberin in plants (Pollard *et al.*, 2008). TAG is generated by connecting fatty acids to a glycerol backbone (Coleman and Lee, 2004). Glycerol-3-phosphate acyltransferases (GPATs) catalyze the first step of TAG biosynthesis by acylating glycerol 3-phosphate at the *sn*-1 or *sn*-2 hydroxyl with an acyl donor, acyl-CoA or acyl-ACP, and generating lysophosphatidic acids (LPAs) that can act as signaling molecules in regulating cell growth (Moolenaar *et al.*, 1997; Sheng *et al.*, 2015; Takeuchi and Reue, 2009). Because GPAT displays the lowest specific activity toward a very broad group of substrates, it has been considered to be the rate-limiting enzyme (Wendel *et al.*, 2009; Zheng and Zou, 2001). In animals, GPATs usually acylate glycerol-3-phosphate at the *sn*-1 position, and are required for membrane lipid synthesis and energy storage. In contrast, in land plants, most GPATs are *sn*-2 GPATs, which catalyze the reaction in which glycerol is an anchor point for the linear or cross link with fatty acids, playing important roles in the assembly of cutin or suberin in plants (Pollard *et al.*, 2008; Yang *et al.*, 2012).

Arabidopsis has eight *sn*-2 GPATs with different functions (Chen *et al.*, 2011*b*). GPAT4 and GPAT8 have high sequence similarity and are suggested to be functionally redundant duplicated genes. Neither the *gpat4* nor the *gpat8* single mutant showed any obvious cuticle defect, whereas the *gpat4gpat8* double mutant exhibited a marked decrease in cutin content in leaves and stems (Li *et al.*, 2007). *GPAT6* is highly expressed in flowers (Zheng *et al.*, 2003). Its mutant displayed defective nanoridges on petal surfaces and a significant reduction of cutin monomers in flowers (Li-Beisson *et al.*, 2009). Further biochemical analyses demonstrated that GPAT4, GPAT6, and GPAT8 prefer C16:0 and C18:1 ω-oxidized substrates and have additional phosphatase activity, resulting in the conversion of *sn*-2 LPA to *sn*-2 MAG, which is also an important intermediate for polyester assembly (Yang *et al.*, 2012). GPAT5 is required for the synthesis of suberin in seed coat and root, and the *gpat5* mutant exhibited strong reduction of very long chain (C22–C24) fatty acid monomers and their derivatives (Beisson *et al.*, 2007). GPAT7, which is phylogenetically most closely related to GPAT5, takes part in suberin synthesis in the wounding response (Yang *et al.*, 2012). GPAT5 and GPAT7 accommodate a broad chain length range of both ω-oxidized and unsubstituted substrates, but they do not possess phosphatase activity. GPAT1, which is mainly expressed in flowers and siliques, also has *sn*-2 acyltransferase activity utilizing both substituted and unsubstituted substrates, but has no phosphatase activity (Zheng *et al.*, 2003). There is no report yet on the function of GPAT2 or GPAT3 (Yang *et al.*, 2012). Notably, GPAT1 and GPAT6 are essential for male plant fertility. Both *gpat1* and *gpat6* mutants display altered endoplasmic reticulum (ER) profiles in tapetal cells, as well as severely reduced pollen production and decreased pollen pollination (Li *et al.*, 2012; Zheng *et al.*, 2003). The *gpat1gpat6* double mutant exhibited short filaments, defective callose degeneration and microspore release, and complete male sterility (Li *et al.*, 2012). Through database searching, we found 17 GPATs in rice. So far, there is only one report of a rice plastidial GPAT on its substrate selectivity and association with chilling tolerance (Zhu *et al.*, 2009). The involvement of GPATs in rice fertility, particularly anther and pollen development, remains unknown.

In this study, we report the functional analysis of an ER-localized GPAT, Glycerol-3-Phosphate Acyltransferase 3 (OsGPAT3), which plays a crucial role in rice male fertility. The *osgpat3* mutant exhibits abnormal tapetum development and defective anther cuticle and pollen exine formation, which is concomitant with a dramatic reduction in aliphatic contents, as well as the down-regulation of genes involved in lipid metabolism and regulation of anther development. We demonstrate that this monocot GPAT plays different roles in male reproduction from its dicot counterpart, providing new insights into the function of glycerolipid biosynthetic enzymes in male fertility.

## Materials and methods

### Plant materials and growth conditions

Rice (*Oryza sativa* L.) plants used in this study were in the 9522 background (*japonica*) and were grown in the paddy field of Shanghai Jiao Tong University. The F2 mapping population was generated from a cross between *osgpat3* (*japonica*) and GuangLuAi 4 (wild type, *indica*) for gene mapping. Male sterile plants in the F2 population were chosen for gene mapping.

### Characterization of mutant phenotype

Plants or flowers were photographed with a Nikon D90 digital camera and a Leica MZ16FA microscope. Observation of anther development by semi-thin sections and transmission electron microscopy were performed as described by Li *et al.* (2006). Anther staging was defined as described previously (Zhang *et al.*, 2011; Zhang and Wilson, 2009). Scanning electron microscopy and analyses of anther waxes, cutin, and internal soluble lipids were performed as described by Shi *et al.* (2011).

### *Map-based cloning of the* OsGPAT3 *gene*

For fine mapping of the *OsGPAT3* locus, bulked segregation analysis was used to identify markers linked to *OsGPAT3* as described by Liu *et al.* (2005). The primer sequences for InDel markers are shown in Supplementary Table S1 at *JXB* online. The *OsGPAT3* locus was first mapped between two InDel molecular markers, CH1132 and RM6094, on chromosome 11. Then, 3000 F2 offspring from the mapping cross were generated, and five InDel markers (ML2, ML4, ML12, AZ11-2, and YUN115.1) were used. *OsGPAT3* was finally defined between InDel markers ML4 and ML12, within a 122 kb region. PCR was performed according to Chu *et al.*, (2006). The PCR products were separated on 6% polyacrylamide denaturing gels, and bands were visualized by a silver-staining method (Liu *et al.*, 2005). Sequence data for the genomic DNA and mRNA of *OsGPAT3* can be found in the NCBI GenBank (https://www.ncbi.nlm.nih.gov/genbank/) and Rice Genome Annotation Project (RGAP; http://rice.plantbiology.msu.edu/) databases under accession numbers Os11g0679700/NM_001074987 and LOC_Os11g45400, respectively.

### *Complementation of the* osgpat3 *mutant*

For functional complementation, the *OsGPAT3* 1.632 kb coding DNA sequence (CDS) and the upstream 3 kb promoter were amplified and subcloned into a modified binary vector *pCAMBIA1301* with the GUS coding region replaced by an eGFP fragment, which carries a hygromycin resistance marker, using the restriction endonucleases *Sac*I and *Spe*I. Calluses induced from young panicles of the homozygous *osgpat3* plants were used for transformation with *Agrobacterium tumefaciens* (EHA105) carrying the *pCAMBIA1301-OsGPAT3-GFP* plasmid or the control plasmid *pCAMBIA1301-GFP* (Li *et al.*, 2006). For transgenic plants, at least 15 independent lines were obtained for each construct. Transgenic plants were identified by PCR using the primers listed in Supplementary Table S1.

### Phylogenetic analysis

Multiple protein sequence alignments were performed using MUSCLE 3.6 (http://www.ebi.ac.uk/Tools/msa/muscle/). A phylogenetic tree was constructed with the alignment of GPAT-like protein sequences of rice, Arabidopsis, and other species. MEGA 4.0 (http://www.megasoftware.net/index.html) and the neighbor-joining method were used, with Poisson correction, pairwise deletion, and bootstrap (1000 replicates; random seed).

### *Quantitative reverse-transcription real-time PCR assay and* in situ *hybridization*

Total RNA was isolated from various rice tissues, including anthers at different developmental stages, using Trizol reagent (Invitrogen) as described by the supplier. An aliquot of 1 μg RNA per sample was used to synthesize cDNA, using a PrimeScript RT reagent Kit with gDNA eraser (Takara). Quantitative reverse-transcription real-time PCR (qRT-PCR) was performed on a Bio-Rad C1000 machine using Takara SYBR Premix Ex Taq^TM^ GC with a standard two-step protocol, consisting of 95 °C for 30s followed by 40 cycles of 95 °C for 5s and 60 °C for 30s. The expression level of *OsACTIN1* was used as an internal control, and a relative quantitation method (Δ cycle threshold) was used to quantify the relative expression level of target genes. Three biological replicates with three technique replicates each were included for statistical analysis and error range analysis. The GenBank accession numbers of the genes used in the qRT-PCR assay are *DPW* (Os03g0167600), *CYP703A3* (Os08g0131100), *CYP704B2* (Os03g0168600), *TDR* (Os02g0120500), *TIP2* (Os01g0293100), and *MTR1* (Os02g0491300) (see Supplementary Table S1 for primer sequences). RNA *in situ* hybridizations were performed as described by Li *et al.* (2006). A 269 bp cDNA fragment of *OsGPAT3* was used for making antisense and sense probes (Supplementary Table S1).

### Subcellular localization of OsGPAT3

The full-length *OsGPAT3* CDS and *OsGPAT3*ΔN were cloned into the *Xho*I and *Spe*I sites of the *pA7-35S::GFP* plasmid. The resulting plasmids were coupled with gold particles and bombarded into onion epidermal cells, which were observed as previously described by Liu and Mehdy (2007). A laser scanning confocal microscope (Leica TCS SP5) was used for the analysis. GFP fluorescent signals were imaged at the excitation wavelength of 488 nm and the emission wavelength of 505–530 nm.

## Results

### *Phenotypic analysis of* osgpat3

**Fig. 1.**
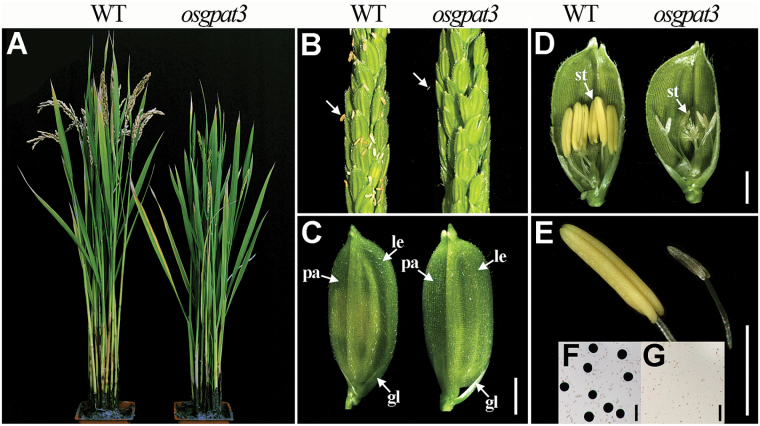
Phenotypic comparison between wild-type (WT) and *osgpat3* mutant rice. (A) A WT plant (left) and an *osgpat3* mutant plant (right) after producing seeds. (B) A WT panicle (left) and an *osgpat3* mutant panicle (right) at the heading stage. The arrows indicate anthers. (C) A WT spikelet (left) and an *osgpat3* mutant spikelet (right). gl, glume; le, lemma; pa, palea. (D) A WT spikelet (left) and an *osgpat3* mutant spikelet (right) after removing half of the lemma and palea. st, stamen. (E) A WT anther (left) showing normal yellow color and an *osgpat3* mutant anther (right), which is smaller and pale to white in color. (F) WT pollen grains stained with 1% I_2_-KI solution at stage 12, showing mature pollen grains. (G) Pollen grains of *osgpat3* stained with 1% I_2_-KI solution at stage 12; no mature pollen grains are present. Bars=2 mm in (C–E) and 50 μm in (F) and (G). (This figure is available in colour at *JXB* online.)

To understand the molecular basis of control of rice male fertility, a completely male-sterile mutant was isolated from our rice mutant library (Chen *et al.*, 2006). This mutant was named *osgpat3* because of a deletion of the putative GPAT gene detected in the mutant by a map-based cloning approach (see below). All the F1 progeny from the backcross between the wild type and *osgpat3* displayed the wild-type phenotype, and the F2 progeny had an approximate 3:1 segregation ratio of wild-type (fertile) and mutant (sterile) phenotypes (310:105, *χ*^2^=0.013, *P*>0.05), suggesting a monofactorial recessive inheritance of the mutation. The mutant exhibited normal vegetative development and inflorescence morphology (Fig. 1A–C), but had pale yellow to white and much smaller anthers compared with those of the wild type (Fig. 1D, E) and lacked mature pollen grains at the late stages of anther development (Fig. 1F, G); these observations indicated that OsGPAT3 is required for anther development and pollen formation in rice.

**Fig. 2.**
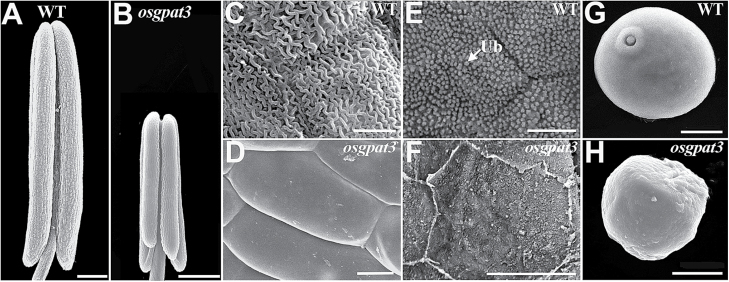
Appearance of the anther and pollen grain in wild-type (WT) and *osgpat3* mutant rice under scanning electron microscopy. (A) WT and (B) *osgpat3* anthers at stage 12 of development. (C, D) The outermost surface of the epidermis of (C) WT and (D) *osgpat3* anthers at stage 12. (E, F) The inner surface of the anther wall layers of (E) WT and (F) *osgpat3* anthers at stage 10. Ub, Ubisch bodies. (G, H) Pollen grains of (G) WT and (H) *osgpat*3 at stage 9. Bars=200 μm in (A) and (B), 5 μm in (C–F), and 10 μm in (G) and (H).

Scanning electron microscopy was used to further observe the phenotypic changes in *osgpat3* at various anther developmental stages as defined previously by Zhang and Wilson (2009). At stage 9, there was no observable anther epidermal difference between wild type and *osgpat3* (data not shown). At stage 10, dense array of granular Ubisch bodies were observed on the inner locule surface of wild-type anthers but not on that of *osgpat3* (Fig. 2E, F). At stage 12, the outer surface of the wild-type anther was covered by well-formed cutinized nanoridges, while the *osgpat3* anther surface was quite smooth and cutinized nanoridges were absent (Fig. 2A–D). In addition, normal pollen grains appeared in wild-type anthers at stage 9, while shrunken and irregularly shaped pollen grains, which became completely aborted at the later stages, were observed in *osgpat3* (Fig. 2G, H). These results showed that mutation of *OsGPAT3* influences the development of the anther cuticle and Ubisch body, and pollen formation.

**Fig. 3.**
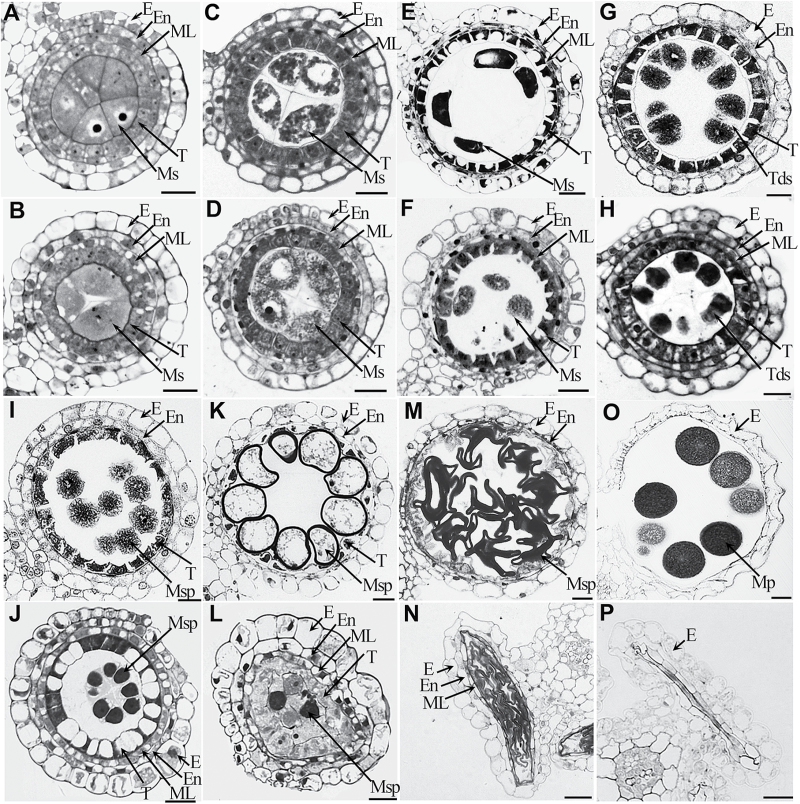
Transverse section analysis of anther development in wild-type and *osgpat3* mutant rice. Locules from the anther section of wild-type (A, C, E, G, I, K, M, O) and *osgpat3* (B, D, F, H, J, L, N, P) from stage 6 to stage 12 of development in sequence (6, 7, 8a, 8b, 9, 10, 11, 12, respectively). E, epidermis; En, endothecium; ML, middle layer; ; Mp, mature pollen Ms, microsporocyte; Msp, microspores; T, tapetum; Tds, tetrads. Bars=15 μm.

Similarly, light microscopic examination of transverse sections of anthers showed that there were no remarkable morphological differences between the wild type and *osgpat3* during the early developmental stages (Fig. 3A–D). At stage 8a, ellipsoidal dyads were formed and tapetal cells became vacuolated and shrunken with darkly stained cytoplasm in the wild type (Fig. 3E). In the *osgpat3* mutant, although dyads were formed, the tapetal cells were less vacuolated and less darkly stained (Fig. 3F). At stage 8b, even though *osgpat3* formed normal tetrads, the cells of the middle layer did not degenerate as did those in the wild type (Fig. 3G, H). At stage 9, the wild-type anther had condensed and deeply stained tapetal cells, and young microspores were freely released from the tetrads. By contrast, at the same stage, *osgpat3* had vacuolated tapetal cells, and microspores were still covered with callose and could not be released from the tetrads (Fig. 3I, J). At stage 10, the wild type displayed degenerating tapetum, as well as vacuolated and round microspores, while the *osgpat3* tapetum became swollen and less stained, the microspores started to degrade, and the anther wall started to collapse (Fig. 3K, L). At stage 11, wild-type microspores became falcate in shape and tapetal cells had almost completely degraded into cellular debris, whereas *osgpat3* microspores displayed an irregular strip-shaped appearance, and the anther wall collapsed into the locule (Fig. 3M, N). At stage 12, the wild type produced mature pollen grains in the locule, whereas *osgpat3* had a flat locule without mature pollen (Fig. 3O, P). These results indicated that OsGPAT3 is essential for the timely differentiation and degradation of the tapetum.

**Fig. 4.**
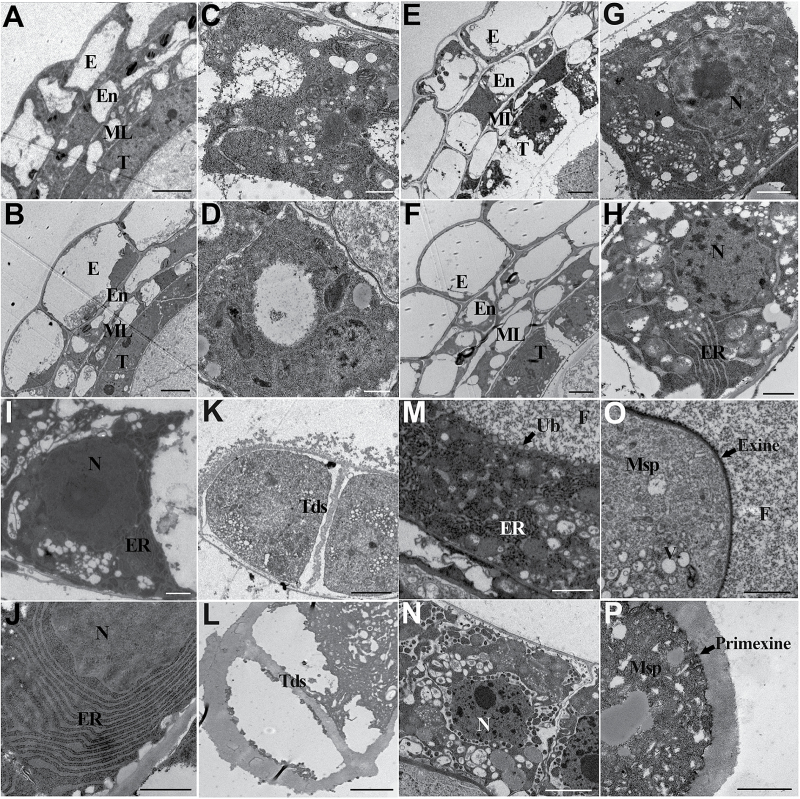
Transmission electron microscopy analysis of anthers in wild-type and *osgpat3* mutant rice. (A, B) Anthers of (A) the wild type and (B) *osgpat3* at stage 6, showing the four layers of the anther wall. (C, D) Tapetal cells of (C) the wild type and (D) *osgpat3* at stage 6. (E, F) Anthers of (E) the wild type and (F) *osgpat3* at stage 8a. (G, H) Tapetal cells of (G) the wild type and (H) *osgpat3* at stage 8a. (I, J) Tapetal cells of (I) the wild type and (J) *osgpat3* at stage 8b. Obviously increased and swollen ER can be observed in *osgpat3*. (K, L) Tetrads in (K) the wild type and (L) *osgpat3* at stage 8b. (M, N) Tapetal cells in (M) the wild type and (N) *osgpat3* at stage 9. The arrow in (M) indicates an Ubisch body (Ub). (O, P) Young microspores in (O) the wild type and (P) *osgpat3* at stage 9. Thick callose surrounds the microspore in *osgpat3*. No exine, and only a coarse primexine, is formed in *osgpat3.* E, epidermis; En, endothecium; ML, middle layer; Ms, microsporocyte; Msp, microspores; N, Nucleus; T, tapetum; Tds, tetrads. Bars=5 μm in (A), (B), (E), (F), (K), and (L), 1 μm in (C), (D), (G), (H), (I), and (J), and 2 μm in (M–P).

To better understand the developmental abnormalities of anther and pollen in the *osgpat3* mutant, transmission electron microscopy was applied. Consistent with the findings of light microscopy of transverse sections, no obvious ultrastructural morphological alteration was observed in *osgpat3* at the early stages of anther development (Fig. 4A–D). At stage 8a, the *osgpat3* mutant tapetal cells showed less stained cytoplasm, and contained swollen ER and more vacuoles and lipidosomes compared with the wild type (Fig. 4E–H). At stage 8b, *osgpat3* tapetal cells displayed markedly increased and expanded ER (Fig. 4I, J). More tapetal cells with increased ER profiles are shown in Supplementary Fig. S3. Consistent with the defective release of microspores from the tetrad, a thicker callose wall surrounding the tetrad was observed in *osgpat3* (Fig. 4K, L). At stage 9, the wild-type tapetum became condensed, and proliferated ER appeared in the tapetal cells (Fig. 4M). In contrast, at this stage almost no ER but abundant vacuoles and lipidosomes were observed in *osgpat3* tapetal cells (Fig. 4N). Additionally, in wild-type anthers the secretory structures Ubisch bodies were seen on the surface of the tapetal cells, fine fibrillar materials released from the tapetum were distributed uniformly throughout the locule, and pollen exine was formed on the surface of the microspores, whereas in the *osgpat3* mutant no Ubisch bodies or fibrillar material, and only coarse primexine, was evident (Fig. 4M–P). These observations confirmed that mutation of *OsGPAT3* affects the development of tapetum and Ubisch bodies, pollen exine formation, and also callose wall degradation.

### *Changes in aliphatic composition in* osgpat3

**Fig. 5.**
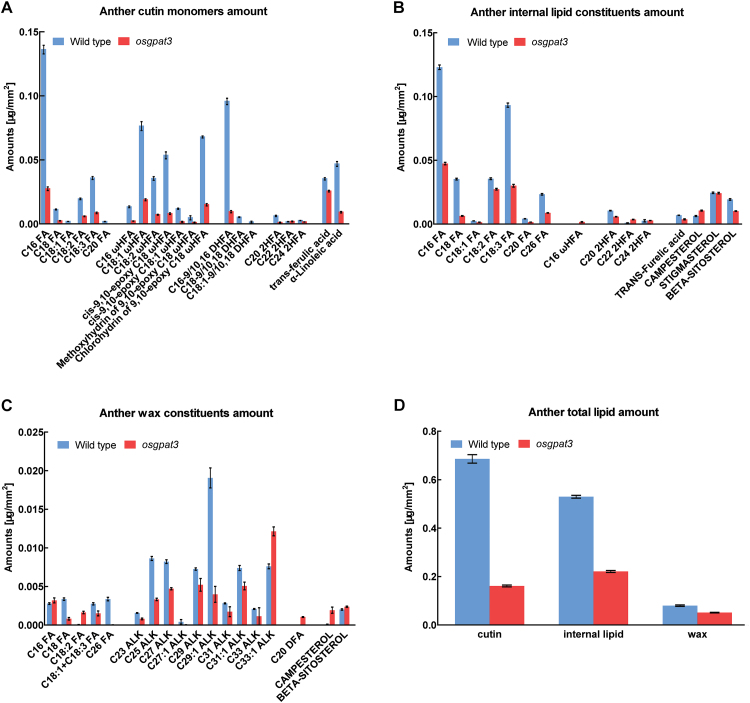
Analysis of lipid content in wild-type and *osgpat3* mutant anthers. Amount per unit surface area (mg mm^–2^) of (A) cutin monomers, (B) internal lipid constituents, (C) wax constituents, and (D) total anther lipid contents in wild-type and *osgpat3* anthers. Error bars indicate SD (*n*=4). Compound names are abbreviated as follows: C16 FA, palmitic acid; C18 FA, stearic acid; C18:1 FA, oleic acid; C18:2 FA, linoleic acid; C18:3 FA, linolenic acid; C20 FA, eicosanoic acid; C26 FA, cerotic acid; ALK, alkane; C16 ωHFA, 16-hydroxy-hexadecanoic acid; C18:1 ωHFA, 18-hydroxy-oleic acid; C18:2 ωHFA, 18-hydroxy-linoleic acid; *cis*-9,10-epoxy C18 ωHFA, *cis*-9,10-epoxy 18-hydroxy-stearic acid; *cis*-9,10-epoxy C18:1 ωHFA, *cis*-9,10-epoxy 18-hydroxy-oleic acid; methoxyhydrin of 9,10-epoxy C18 ωHFA, methoxyhydrin of 9,10-epoxy 18-hydroxy-stearic acid; chlorohydrin of 9,10-epoxy C18 ωHFA, chlorohydrin of 9,10-epoxy 18-hydroxy-stearic acid; C16-9/10, 16 DHFA, 9(10), 16-dihydroxy-hexadecanoic acid; C18-9/10, 18 DHFA, 9(10), 18-dihydroxy-stearic acid; C18:1–9/10, 18 DHFA, 9(10), 18-dihydroxy-oleic acid; C20 2HFA, 2-hydroxyeicosanoic acid; C20 DFA, eicosane-1, 20-dioic acid; C22 2HFA, 2-hydroxydocosanoic acid; C24 2HFA, 2-hydroxytetracosanoic acid. (This figure is available in colour at *JXB* online.)

The defective anther cuticle and Ubisch body in *osgpat3* suggested that mutation of OsGPAT3 affects anther cuticular lipid profiling. To test this hypothesis, the composition of waxes, cutin, and total soluble lipids in wild-type and *osgpat3* anthers was analyzed by gas chromatography-flame ionization detection (GC-FID) combined with gas chromatography-mass spectrometry (GC-MS). We used the approach described by Li *et al.* (2010) to calculate the surface area of the anthers, in which the calculated values of surface area were plotted against the weight of each sample (Supplementary Fig. S1). Analytical results showed that the total wax, cutin, and total internal lipid levels in *osgpat3* anthers were decreased by 37.5%, 76.8%, and 58.5%, respectively, compared with those of the wild type (Fig. 5D), which was attributed to the significant reduction of almost every lipid molecule in *osgpat3* mutant anthers (Fig. 5A–C). These chemical analysis data not only confirmed the defective anther cuticle and pollen exine patterning observed by scanning and transmission electron microscopy, but also indicated that the mutation of *OsGPAT3* significantly affects the biosynthesis of these lipid compounds, which are required for anther cuticle and pollen exine formation.

### *Map-based cloning and functional complementation of* osgpat3

**Fig. 6.**
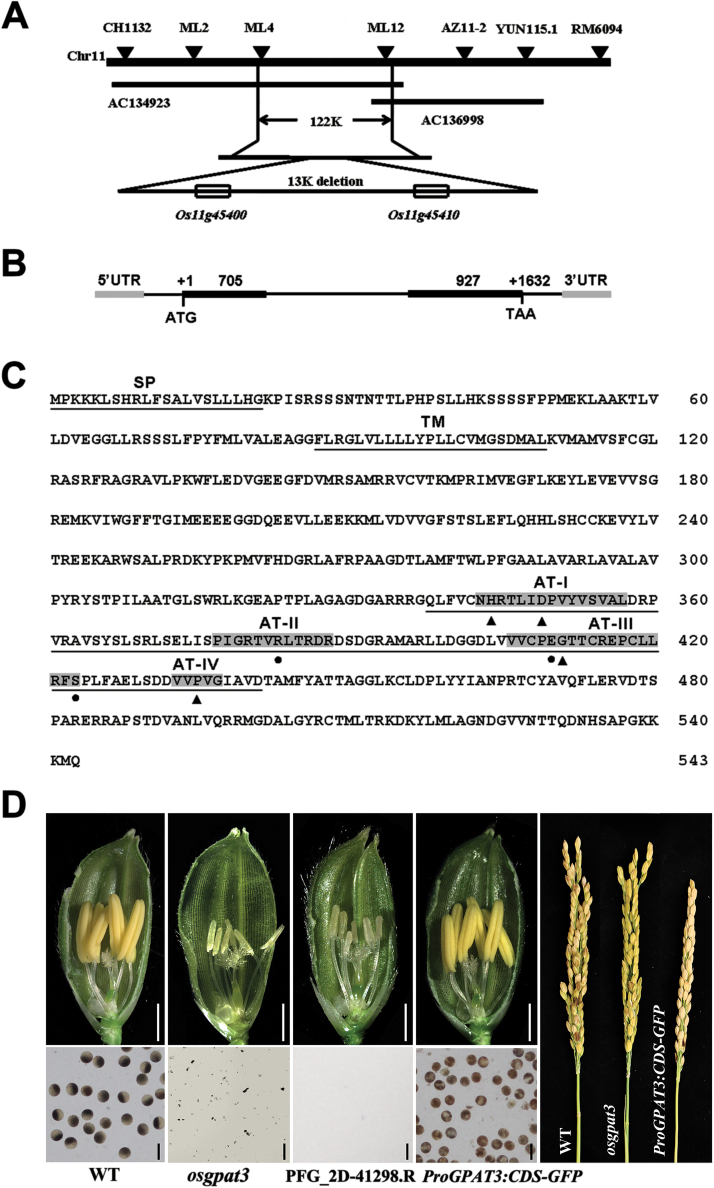
Molecular identification and sequence analysis of OsGPAT3. (A) Fine mapping of the *osgpat3* mutation on chromosome 11. The names and positions of the molecular markers are indicated. AC134923 and AC136998 are genomic DNA accession numbers. The mutation was mapped to a 122 kb region between two molecular markers (ML4 and ML12). (B) A schematic representation of the exon and intron organization of *LOC_Os11g45400*. (C) Predicted protein sequence of OsGPAT3. The protein is predicted to contain a 21 amino acid signal peptide (SP), a transmembrane domain (TM), and a conserved acyltransferase domain (AT) with four conserved motifs. Dots indicate key amino acids important for substrate binding and triangles indicate key amino acids important for catalysis. (D) Functional complementation of *osgpat3* with the full-length CDS of *LOC_Os11g45400* fused to GFP, driven by its own promoter (3 kb). The *LOC_Os11g45400* T-DNA insertion homozygous mutant PFG_2D-41298.R also shows a similar male sterility phenotype to that of the *osgpat3* mutant. WT, wild type. Bars=2 mm for spikelets and 50 μm for pollen grains. (This figure is available in colour at *JXB* online.)

To identify the mutant gene, a map-based cloning approach was used. Through fine mapping, the mutation was located to a 13 kb deletion on chromosome 11. There are two putative genes in the 13 kb deleted region (Fig. 6A). One is *LOC_Os11g45410*, encoding a pentatricopeptide repeat-containing protein (PPR) without any functional characterization; the other gene is *LOC_Os11g45400*, which is presumed to encode a GPAT. To clarify which gene is responsible for the mutant phenotype, genetic complementation was performed in the homozygous *osgpat3* mutant. Transgenic lines with the coding sequence of *LOC_Os11g45400* fused to GFP driven by its own promoter (3 kb) restored male fertility (Fig. 6D). Moreover, a homozygous mutant PFG_2D-41298.R (Jeon *et al.*, 2000) in which the T-DNA fragment was inserted within the second exon of *LOC_Os11g45400*, showed a similar sterile phenotype to *osgpat3* (Fig. 6D). These results confirmed that the deletion of *LOC_Os11g45400* is responsible for the developmental defects observed in *osgpat3* plants. According to the annotation from RGAP, *LOC_Os11g45400* contains two exons and one intron (Fig. 6B), and is predicted to encode a GPAT protein of 543 amino acids, containing a transmembrane domain and an acyltransferase domain, but no phosphatase domain (Fig. 6C; Mañas-Fernández *et al.*, 2010). These results indicated that deletion of the *LOC_Os11g45400* gene is responsible for the defective male fertility in *osgpat3*.

### OsGPAT3 belongs to a monocot-specific clade of the plant *sn*-2 GPAT family

**Fig. 7.**
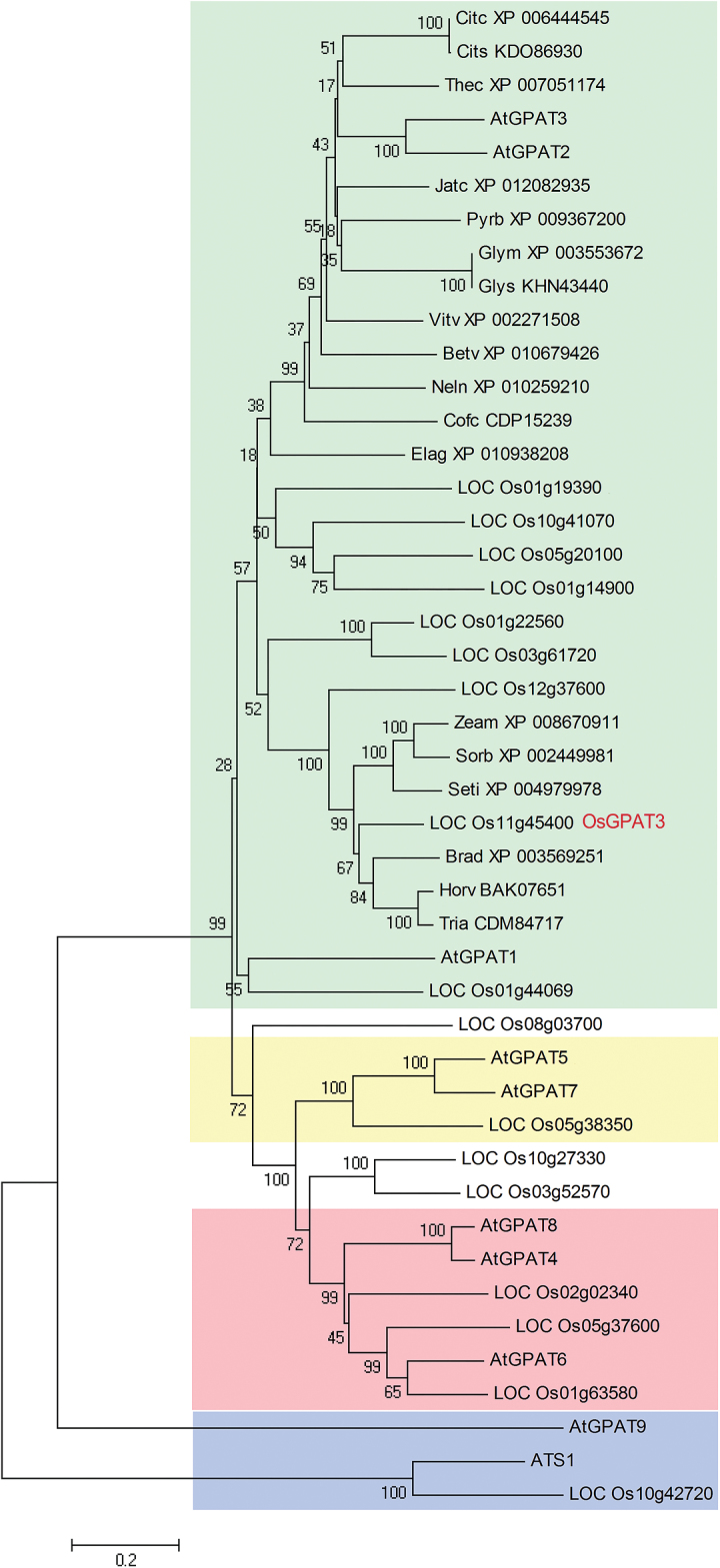
Phylogenetic tree of the GPAT family and OsGPAT3-related orthologs. A neighbor-joining phylogenetic tree was constructed with of all the Arabidopsis and rice GPATs and OsGPAT3 orthologs from other 18 species (E-value less than 7e-132) found in the NCBI, RGAP, and TAIR databases. The proteins are named according to their gene names or NCBI accession numbers. Bootstrap values are percentage of 1000 replicates. The length of the branches is proportional to the amino acid variation rates. Different clades are highlighted in different colors, representing the *sn*-1 GPAT (blue), the GPAT4/6/8 clade (red), the GPAT5/7 clade (yellow), and the GPAT1/2/3 clade (green). At, *Arabidopsis thaliana*; Os, *Oryza sativa*; Citc, *Citrus clementina*; Cits, *Citrus sinensis*; Thec, *Theobroma cacao*; Jatc, *Jatropha curcas*; Pyrb, *Pyrus x bretschneideri*; Vitv, *Vitis vinifera*; Glym, *Glycine max*; Glys, *Glycine soja*; Betv, *Beta vulgaris*; Neln, *Nelumbo nucifera*; Cofc, *Coffea canephora*; Elag, *Elaeis guineensis*; Zeam, *Zea mays*; Sorb, *Sorghum bicolor*; Seti, *Setaria italica*; Brad, *Brachypodium distachyon*; Horv, *Hordeum vulgare*; Tria, *Triticum aestivum*. (This figure is available in colour at *JXB* online.)

To clarify the evolutionary role and potential function of OsGPAT3, we performed phylogenetic analysis by searching the public databases NCBI, RGAP, and TAIR, using BLASTP with the full length of the OsGPAT3 predicted amino acid sequence as a query. Since the family of GPATs is quite large, we only collected a total of 45 protein sequences of all the GPAT members from Arabidopsis and rice, and the top 18 closely related OsGPAT3 homologs from other 18 plant species. Subsequently, we constructed a neighbor-joining phylogenetic tree of the 45 sequences (Fig. 7), which were grouped into two main clades. Rice plastidial GPAT (*LOC_Os10g42720*), together with Arabidopsis plastidial ATS1 and ER-bound AtGPAT9, belonged to the *sn*-1 clade. The *sn*-2 GPAT clade included all the other plant members, which could be mainly classified into three subclades: the AtGPAT4/6/8-related subclade required for the biosynthesis of cutin, the AtGPAT5/7-related subclade associated with the biosynthesis of suberin, and the AtGPAT1/2/3-related subclade, which was less functionally characterized than the other two subclades. *LOC_Os11g45400* was relatively close to *AtGPAT3*, and therefore was named *OsGPAT3*. The *sn*-2 clade is a land-plant-specific lineage associated with the landmark transition from aquatic to terrestrial habitats (Yang *et al.*, 2012). Compared with the first two subclades, the third *sn*-2 GPAT subclade is more divergent and has more members, and is considered to be the evolutionarily more recent clade. So far, only AtGPAT1 in the third subclade has been found to have *in vitro* enzyme activity, and is essential for tapetum differentiation and male fertility (Zheng *et al.*, 2003). No enzyme activity was detected for AtGPAT2 or AtGPAT3, and their mutants showed no obvious phenotype in organs such as leaves, flowers, or seeds (Yang *et al.*, 2012). There are nine rice GPATs in the third subclade. Among them, OsGPAT3 is closer to members from *Brachypodium distachyon*, *Hordeum vulgare*, *Triticum aestivum*, *Zea mays*, *Sorghum bicolor*, *Setaria italica*, and three other members from rice. Based on the fact that *osgpat3* displayed a remarkably defective phenotype in the anther and pollen, we propose that OsGPAT3 and its homologs in rice may have evolved divergently, leading to diversified functions different from those in dicots. Thus, OsGPAT3 may represent one unique *sn*-2 GPAT member specific to monocots.

### OsGPAT3 *is mainly expressed in tapetum and microspores*

**Fig. 8.**
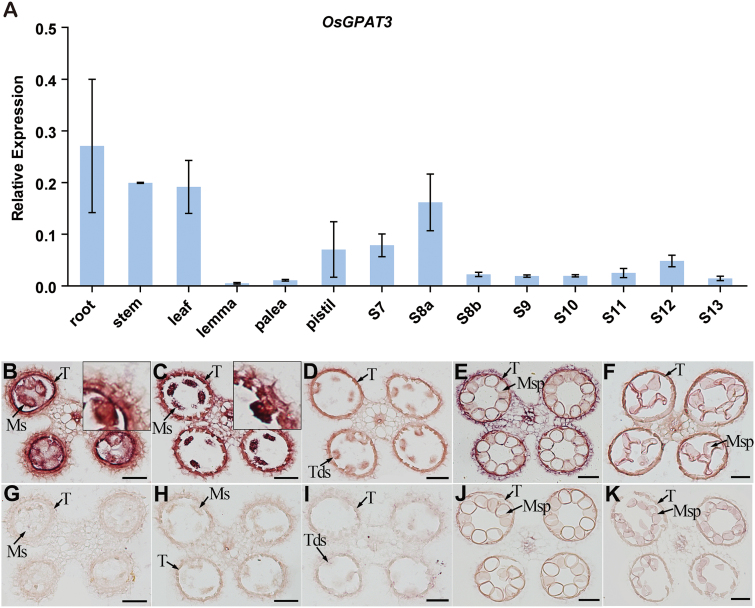
Expression patterns of *OsGPAT3* in wild-type anthers and other tissues. (A) qRT-PCR analysis of the expression level of *OsGPAT3* in wild-type root, stem, leaf, lemma, palea, pistil, and in anthers at different developmental stages (S7–S13). Rice *OsActin1* was used as a normalizer control. Error bars indicate the SE of three biological replicates. (B–K) *In situ* analysis of the expression of *OsGPAT3* in wild-type anthers at stages 7, 8a, 8b, 10, and 11, respectively, with antisense probe (B–F) and sense probe (G–K). The insets in (B) and (C) show an enlarged section. Ms, microsporocyte; Msp, microspores; T, tapetum; Tds, tetrads. Bars=50 μm. (This figure is available in colour at *JXB* online.)

To further understand the function of *OsGPAT3* in male sterility, we investigated the expression pattern of *OsGPAT3* in anthers at various developmental stages and other tissues in wild-type plants using qRT-PCR. In anther tissues, the expression of *OsGPAT3* was detectable as early as stage 7, peaked at stage 8a, declined gradually until stage 10, then increased and peaked again at a lower level at stage 12, and thereafter declined (Fig. 8A). This dynamic expression pattern of *OsGPAT3* in anther tissues was also confirmed by GUS staining (Supplementary Fig. S2A), and was consistent with reported data from the Rice Oligonucleotide Array Database (http://www.ricearray.org/; see Supplementary Fig. S2B). Although relatively higher levels of expression of *OsGPAT3* were also detected in stem, root, and leaf tissues, and moderate expression was detected in pistil, no obvious morphological abnormalities were observed in these organs in *osgpat3* plants, suggesting a possible redundant function of *OsGPAT3*-related homologs in these tissues.

To determine the spatial and temporal expression patterns of *OsGPAT3* more precisely, we performed *in situ* hybridization with wild-type anther sections. The strong signal of *OsGPAT3* transcripts was detected mainly in microspores and tapetum, and weak signals were also detected in the middle layer and the endothecium layer of the anther wall (Fig. 8B–K). The tendency to variation in signal intensity at different stages was also in agreement with the results of qRT-PCR and GUS staining analyses. These results supported that *OsGPAT3* is directly involved in tapetal development, anther development, and pollen formation.

### OsGPAT3 localizes to endoplasmic reticulum

**Fig. 9.**
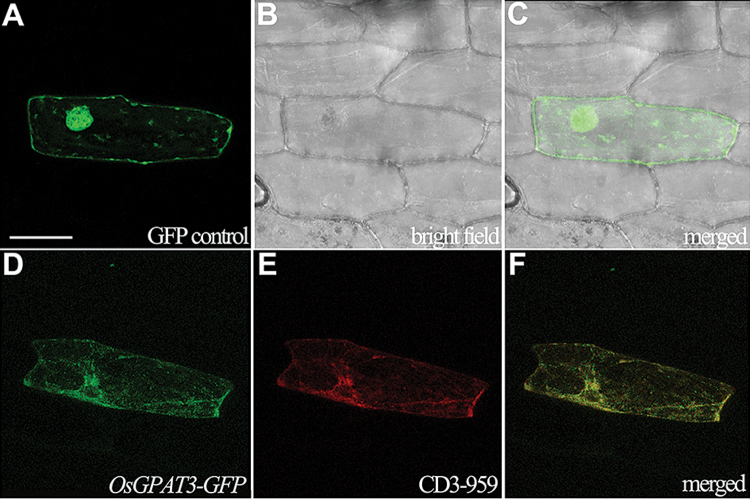
Confocal microscopic images showing the subcellular localization of OsGPAT3-GFP. (A–C) Localization of GFP control (*35S:GFP*) in onion epidermal cells. (D) Localization of OsGPAT3-GFP (*35S:OsGPAT3-GFP*) in onion epidermal cells. (E) Red fluorescence shows the ER localization of an ER marker protein (CD3-959) in the same cell as shown in (D). (F) Merged image of (D) and (E). Bars=100 μm. (This figure is available in colour at *JXB* online.)

OsGPAT3 has a 21 amino acid putative signal peptide at the N-terminus that was predicted to be localized to the mitochondrion or chloroplast (SignalP and TargetP; http://www.cbs.dtu.dk/services/). To validate this prediction and to further understand the function of OsGPAT3, we made three constructs: *OsGPAT3-GFP* containing the full-length cDNA of *OsGPAT3*, *OsGPAT3ΔN-GFP* without the sequence encoding the 21 amino acid peptide, and a GFP control, all driven by the CaMV35S promoter, to determine the subcellular localization of OsGPAT3. When these individual constructs were transformed into onion epidermal cells by particle bombardment, OsGPAT3-GFP signal was observed mainly at the ER (Fig. 9D), mimicking the signal of an ER-localized marker protein (CD3-959, a combination of the AtWAK2 signal peptide and the ER retention signal His-Asp-Glu-Leu; Fig. 9E, F) (Nelson *et al.*, 2007). Although OsGPAT3 lacks the classic ER retention signal KDEL, it has one KKXX motif at the C-terminus and three internal RXR motifs, all of which are also well-defined ER retrieval motifs (Gao *et al.*, 2014; Shikano and Li, 2003). Furthermore, the co-transformation of *OsGPAT3-GFP* with a mitochondrial marker gene did not show overlapping of the fluorescent signals (data not shown), which is consistent with previous reports that there is no mitochondrial localization of Arabidopsis AtGPAT2 or AtGPAT3 (Shen *et al.*, 2003; Zheng *et al.*, 2003). In addition, the *OsGPAT3ΔN-GFP* construct showed the same fluorescent profile as that of *OsGPAT3-GFP* (data not shown), suggesting that the 21 amino acid sequence at the N-terminus of OsGPAT3 was not a signal peptide. These data showed that OsGPAT3 is an ER-localized GPAT.

### *Expression of genes associated with anther development is down-regulated in* osgpat3

**Fig. 10.**
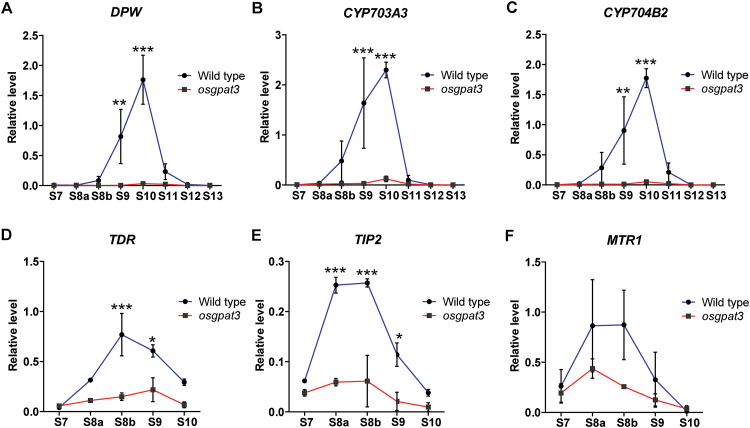
Analysis of the expression of anther development regulatory genes using qRT-PCR at different developmental stages. (A–C) qRT-PCR expression analysis of the cuticle and pollen wall development-related lipid biosynthetic pathway genes (A) *DPW*, (B) *CYP703A3*, and (C) *CYP704B2* in stage 7 to 13 anthers from wild-type and *osgpat3* rice. (D–F) qRT-PCR expression analysis of anther development-related transcription factor genes (D) *TDR* and (E) *TIP2*, and (F) a fasciclin glycoprotein protein-encoding gene, *MTR1*, in stage 7 to 10 anthers from wild-type and the *osgpat3* mutant rice. *OsActin1* was used as a normalizer control. Error bars indicate the SE of three biological replicates. *P<0.05, **P<0.01, ***P<0.001. (This figure is available in colour at *JXB* online.)

Because *osgpat3* showed a dramatic decrease in most kinds of detectable lipid molecules and defects in anther cuticle and pollen exine formation, we hypothesized that *osgpat3* may have impacts on the expression of genes involved in the lipid metabolism required for anther cuticle or exine formation. To test this hypothesis, we compared the expression of a set of genes known to be associated with lipid metabolism in wild-type and *osgpat3* anthers using qRT-PCR. The results showed that the expression of not only the three lipid biosynthetic genes (*DPW*, *CYB704B2*, and *CYP703A3*) (Fig. 10A–C), but also two transcription factors (*TDR* and its interaction protein *TIP2*), which are associated with tapetum degeneration and lipid metabolism (Fig. 10D, E), were significantly down-regulated in the *osgpat3* mutant. In addition, the expression level of MTR1 (Fig. 10F), a secretory fasciclin glycoprotein-encoding gene that affects the development of both tapetum and microspores (Tan *et al.*, 2012), was down-regulated in the *osgpat3* mutant. These results suggested that OsGPAT3 may play important roles in the early stages of anther development.

## Discussion

Lipids, their derivatives, and their pathway-related genes are critical for reproductive development and fertility in higher plants. So far as we know, the tapetal lipid metabolism contributes significantly to anther wall cuticle and pollen exine/tryphine formation during anther development. In tapetal cells, *de novo* synthesized fatty acids were either reduced to fatty alcohols by MS2/DPW in plastid or translocated to ER for activation via ACOS, elongation through long-chain acyl-CoA synthetase (LACS), and hydroxylation by CYP450s (Shi *et al.*, 2015). Glycerol is an essential backbone of plant polyesters such as suberin (Pollard *et al.*, 2008); however, its presence in plant cutin was discovered only comparatively recently (Graça *et al.*, 2002). The linking of fatty acids to glycerol that is catalyzed by GPAT was suggested to occur after oxidization (Beisson *et al.*, 2012; Yang *et al.*, 2012); therefore, various substituted fatty acids, such as ω-hydroxylated fatty acids and α, ω-dicarboxylic fatty acid, are thought to be the substrates of those GPATs. For example, the Arabidopsis AtGPAT6 prefers C16 and C18 ω-oxidized acyl-CoA substrates, while AtGPAT1 can use both unsubstituted and substituted acyl-CoAs.

To answer the question of whether OsGPAT3 works downstream of the previously reported lipid metabolic enzymes, such as DPW, CYP704B2, and CYP703A3, which are involved in the regulation of rice male fertility, we performed an enzyme activity assay of OsGPAT3 with substrates that are the products of DPW, CYP704B2. and CYP703A3 or the dominant components of plant biopolymers. This assay did not provide any positive results (data not shown); this is consistent with findings in Arabidopsis, in which AtGPAT2 or AtGPAT3 also showed no activity to tested fatty acid-CoA substrates (Yang *et al.*, 2012; Zheng *et al.*, 2003). It is possible that an appropriate substrate has not yet been tested or that unidentified factors are missing from the assay system, and these possibilities deserve further investigation in the future. However, the significantly altered cuticular lipid profile in *osgpat3* mutant anthers, together with the high amino acid sequence similarity of OsGPAT3 to other known GPAT family members, suggests that OsGPAT3 is an active enzyme that is required for male fertility in rice. In addition, our comparative gene expression analysis through the Rice Oligonucleotide Array Database found that the expression of *OsGPAT3* peaks earlier than that of *DPW*, *CYP704B2*, and *CYP704A3*, consistent with previous studies (Li *et al.*, 2010; Shi *et al.*, 2011; Yang *et al.*, 2014) and our qRT-PCR data (Fig. 8A; Supplementary Fig. S2B). Combining the findings that the expression of *DPW*, *CYP704B2*, and *CYP703A3*, and of tapetum programmed cell death regulatory genes such as *TDR* and *TIP2*, were significantly down-regulated in the *osgpat3* mutant, we propose that OsGPAT3 may not work directly downstream of DPW, CYP704B2, or CYP703A3. Rather, OsGPAT3 may affect male fertility via its function at earlier developmental stages, with a mechanism that differs from that of these previously reported lipid metabolic genes, which are usually expressed at relatively late anther developmental stages. It is possible that a gene like *LOC_Os1g63580*, a homolog of *AtGPAT6*, is likely to work downstream of *DPW*, *CYP704B2*, and *CYP704A3* (Supplementary Fig. S2B).

In Arabidopsis, AtGPAT1 and AtGPAT6 were reported to be required for male fertility. The *atgpat1* mutant showed perturbed tapetum degeneration and massive arrest of pollen development, and the *atgpat6* mutant exhibited large-scale abortion of pollen grains and defective pollen wall formation. Both *atgpat1* and *atgpat6* single mutants were semi-sterile and displayed reduced ER proliferation in the tapetal cells, while the *atgpat1atgpat6* double mutant showed a defect of microspore release from tetrads and complete male sterility (Li *et al.*, 2012; Zheng *et al.*, 2003). Our results showed that loss of function of *OsGPAT3* expression in tapetum and microspores disrupts tapetum development and metabolism, leading to defective anther cuticle and pollen exine formation (including the absence of Ubisch bodies), and eventual complete abortion of pollen grains. Unlike *atgpat1* or *atgpat6*, the *osgpat3* mutant exhibited increased ER expansion and proliferation in tapetal cells at anther developmental stage 8, and almost complete absence of ER but abundant vacuoles and lipidosomes (probably generated from the degradation of ER) at stage 9, indicating that the lesion of OsGPAT3 may lead to abnormal ER development and degradation, which may affect tapetum metabolism and function. This functional difference between OsGPAT3 and AtGPAT1/6 may reflect structural differences in tapetum; the tapeta of rice and other cereals exhibit characteristic orbicules/Ubisch bodies, which have not been observed in members of the Brassicaceae family, including Arabidopsis, which have unique secretory tapeta containing specialized organelles including elaioplasts and tapetosomes (Zhang *et al.*, 2011).

It was reported that the land plant GPATs belong to a *sn*-2 GPAT family that differs from animal GPATs (Yang *et al.*, 2012). Clade classification of this family reflected their biochemical functions, which are evolutionally associated with the plants’ adaptation during the transition from aquatic to terrestrial habitats. While the first and second clades are conserved among the tracheophytes in the biosynthesis of cutin or suberin, the function of the third clade remains poorly known. Our phylogenetic analysis showed that the monocotyledon subclade to which OsGPAT3 belongs includes three other rice members with unknown function (Fig. 7). While *atgpat2* or *atgpat3* mutants showed no obvious macroscopic or chemical phenotype (Yang *et al.*, 2012), the *osgpat3* mutant showed significant changes in anther cuticular lipid profiling and was male sterile, suggesting that OsGPAT3 plays an indispensable role in male reproduction that is distinct from the role of its dicot counterpart. Further functional characterization of the third land plant *sn*-2 GPAT clade will facilitate our understanding of the evolutionary and molecular aspects of the grass GPAT proteins in plant evolution and adaptation.

In conclusion, we identified a land plant *sn*-2 GPAT family member, *OsGPAT3*, in rice, which encodes an ER-localized GPAT. The dysfunction of OsGPAT3 significantly affects the anther cuticle and pollen exine formation, and leads to eventual male sterility. This work has expanded our understanding of lipid metabolism, particularly the poorly known glycerolipid metabolism, in plant male reproductive development. The conservation and divergence of the *sn*-2 GPAT family in land plants merit further investigations, which would help us to explore the evolutionary and biochemical functions of lipid metabolism in plant fertility.

## Supplementary data

Supplementary data are available at *JXB* online.

Fig. S1. The ratio of weight/surface area of the anthers in the wild type and *osgpat3* mutant.

Fig. S2. Expression patterns of *OsGPAT3* in wild-type anthers.

Fig. S3. Transmission electron microscopy analysis of tapetal cell development in the wild type and *osgpat3* mutant at stage 8.

Table S1. Primer sequences used in this study.

## Author contributions

DZ and WL conceived the original screening and research plans; DZ and WL supervised the project and experiments; XM performed most of the experiments; QZ and GL participated in primer design, vector construction, and lipid analysis; JS, SQ, LZ, ZL, and MC provided technical assistance to XM; JS and XM conceived and wrote the paper, with contributions from all the authors; DZ supervised and complemented the writing of the paper.

## Supplementary Material

supplementary_figures_S1_S3_table_S1
