# Supplementary material for: Glycerol-3-Phosphate Acyltransferase 3 (OsGPAT3) is required for anther development and male fertility in rice
Source: J Exp Bot. 2017 Jan 12;68(3):513–26. doi: 10.1093/jxb/erw445 (PMC6055571; doi:10.1093/jxb/erw445)
Supplement: supplementary_figures_S1_S3_table_S1 [file erw445_suppl_supplementary_figures_s1_s3_table_s1.pdf]

Glycerol-3-Phosphate Acyltransferase 3 (OsGPAT3) is required for anther development and male fertility in rice

Authors:

Xiao Men, Jianxin Shi, Wanqi Liang, Qianfei Zhang, Gaibin Lian, Sheng Quan, Lu Zhu, Zhijing Luo, Mingjiao Chen, and Dabing Zhang

Figure S1. The ratio of weight/surface area of the anthers in the wild type and *osgpat3* mutant.

Figure S2. Expression patterns of *OsGPAT*3 in wild type anthers. A, GUS staining of *ProGPAT3:GUS* spikelets at different stages. Bars = 2 mm. B, Heatmap expression data of *OsGPAT3*, *DPW*, *CYP704B2*, *CYP703A3* and *LOC_Os01g63580*, a homolog of *AtGPAT6*, during anther development (Affymetrix GSE 13988, Rice Oligonucleotide Array Database: http://www.ricearray.org). An1, hypodermal archesporial cells forming stage; Mei1, pollen mother cells at pre-meiotic S/G2 stage; M1, pollen mother cells at meiotic leptotene stage; M2, pollen mother cells at meiotic zygotene-pachytene stage; M3, pollen mother cells at meiotic diplotene-tetrad stage; P1, uni-nucleated gametopyte stage; P2, bi-cellular gametopyte stage; P3, tri-cellular mature pollen stage.

Figure S3. Transmission electron microscopy analysis of tapetal cells development in the wild type and *osgpat3* mutant at stage 8. A-D, tapetal cells of the wild type at stage 8. E-H, tapetal cells of the *osgpat3* mutant at stage 8. Obviously increased and swollen ER can be observed in the tapetal cells of *osgpat3* mutant. N, Nucleus; ER, endoplasmic reticulum. Bars = 1 μm in (A), (C), (E), (G) and 2 μm in (B), (D), (F), (H).

Table S1. Primer sequences used in this study.

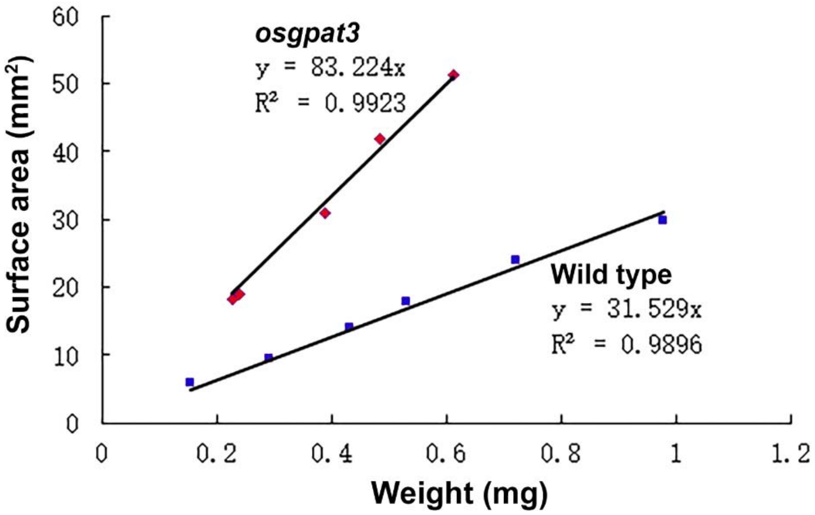

Figure S1. The ratio of weight/surface area of the anthers in the wild type and *osgpat3* mutant.

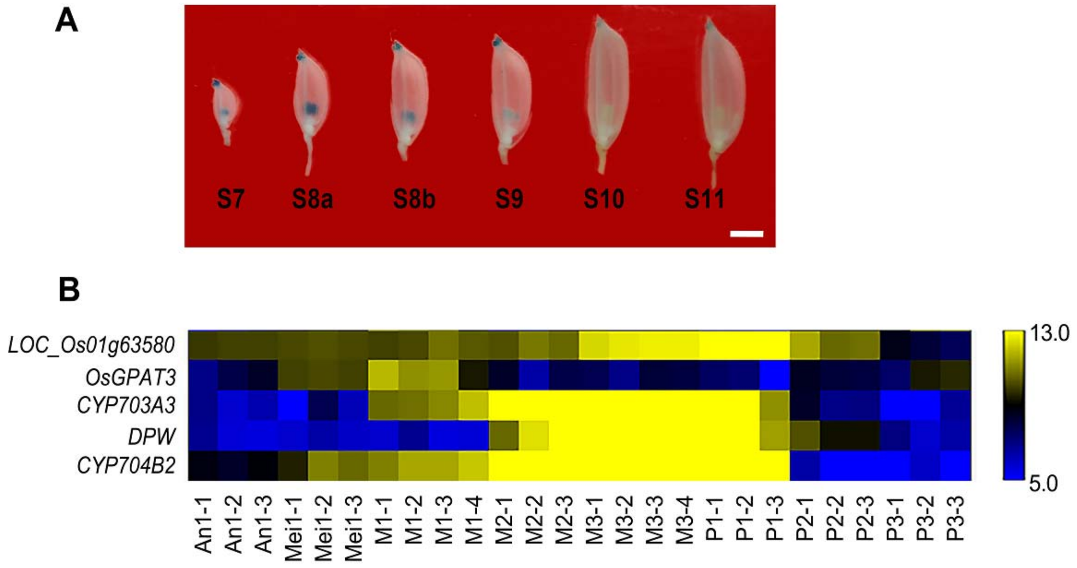

Figure S2. Expression patterns of *OsGPAT*3 in wild type anthers. A, GUS staining of *ProGPAT3:GUS* spikelets at different stages. Bars = 2 mm. B, Heatmap expression data of *OsGPAT3*, *DPW*, *CYP704B2*, *CYP703A3* and *LOC_Os01g63580*, a homolog of *AtGPAT6*, during anther development (Affymetrix GSE 13988, Rice Oligonucleotide Array Database: http://www.ricearray.org). An1, hypodermal archesporial cells forming stage; Mei1, pollen mother cells at pre-meiotic S/G2 stage; M1, pollen mother cells at meiotic leptotene stage; M2, pollen mother cells at meiotic zygotene-pachytene stage; M3, pollen mother cells at meiotic diplotene-tetrad stage; P1, uni-nucleated gametopyte stage; P2, bi-cellular gametopyte stage; P3, tri-cellular mature pollen stage.

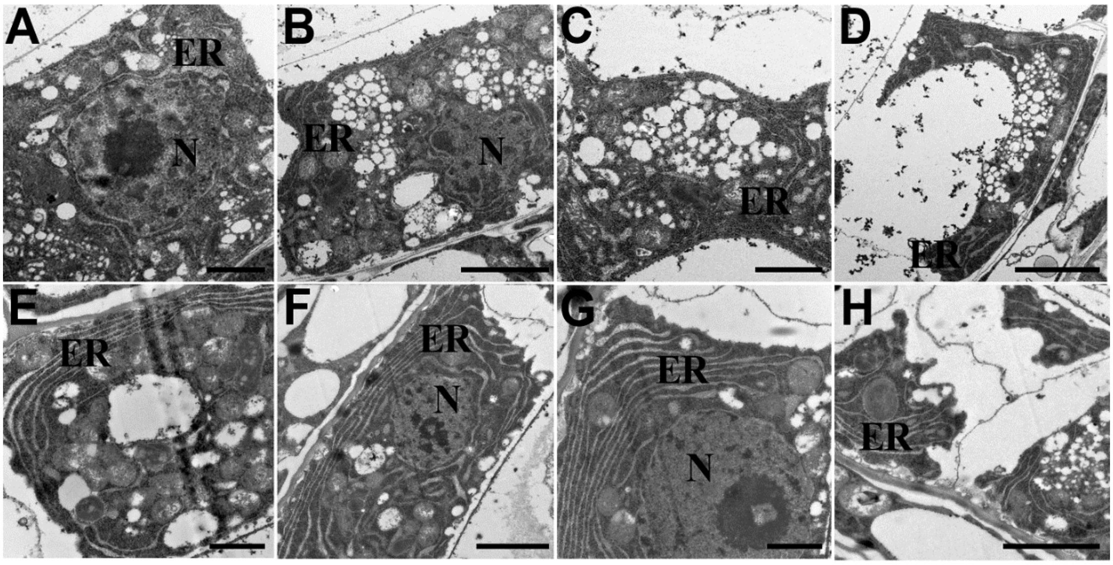

Figure S3. Transmission electron microscopy analysis of tapetal cells development in the wild type and *osgpat3* mutant at stage 8. A-D, tapetal cells of the wild type at stage 8. E-H, tapetal cells of the *osgpat3* mutant at stage 8. Obviously increased and swollen ER can be observed in the tapetal cells of *osgpat3* mutant. N, Nucleus; ER, endoplasmic reticulum. Bars = 1 μm in (A), (C), (E), (G) and 2 μm in (B), (D), (F), (H).

Table S1. Primer sequences used in this study.

Primer name	Sequence (5'-3')	Annotation
pA7-OsGPAT3-F	CCCTCGAGATGCCCAAGAAGAAGCTGTCACA	subcellular-localization
pA7-(ΔN)OsGPAT3-F	CCCTCGAGAAGCCTATCTCTAGATCATCATCCAACAC	subcellular-localization
pA7-OsGPAT3-R	GGACTAGTACTTGCATCTTCTTCTTCCCCGGAG	subcellular-localization
OsGPAT3pro-F	CCGAGCTCGACCATATGAGTGCATAGAAATTAATGG	GUS staining
OsGPAT3pro-R	AACCATGGGGATCCGGTGTACTGTTGTTGGTGGCAA	GUS staining
p1300+OsGPAT3eGFP-F	CGGGATCCATGCCCAAGAAGAAGCTGTCACAT	complementary expression in *osgpat3*
p1300+OsGPAT3eGFP-R	GGACTAGTTTGCATCTTCTTCTTCCCCGGAG	complementary expression in *osgpat3*
2D-41298-F	CCTCATGTGCATTGCTGTATG	genotyping
2D-41298-R	GGGTTGGCGATGTAGTAGAG	genotyping
JD-HYG-F	GATGTTGGCGACCTCGTATT	genotyping
JD-HYG-R	GAGCCTGACCTATTGCATCTC	genotyping
CH1132-F	ATCGGCTACTCATCCACCT	mapping
CH1132-R	TTTTGGGACGAAGGGAG	mapping
ML2-F	AGCTGTTACCGCAGTCGA	mapping
ML2-R	TGACGTGTGTAAAATTTGTGT	mapping
ML4-F	GCCTCTGTGATGTTGTCGAG	mapping
ML4-R	GGATCAATTGGGAGAAGGTG	mapping
ML12-F	GGCTTCTCCTAGGGCTTACT	mapping
ML12-R	ACACCAACATGAGAACAACC	mapping
YUN115.1-F	TGCGAATTGTTCCGGTTAT	mapping
YUN115.1-R	AAATGTCGCATGTAACCACA	mapping
RM6094-F	TGCTTGATCTGTGTTCGTCC	mapping
RM6094-R	TAGCAGCACCAGCATGAAAG	mapping
OsActin-qRT-F	CGATAACAGCTCCTCTTGGC	qRT-PCR
OsActin-qRT-R	GAGATCACTGCCTTGGCTCC	qRT-PCR
OsGPAT3-qRT-F	TTCACTGGCATCATGGAGGAGGAA	qRT-PCR

Primer name	Sequence (5'-3')	Annotation
OsGPAT3-qRT-R	AACACCATCGGCTTCGGGTACTT	qRT-PCR
DPW-qRT-F	GTAGTCGGAGATGTCAGAGAAGCC	qRT-PCR
DPW-qRT-R	GATCCCTCCAGGTGCTCTCG	qRT-PCR
CYP703A3-qRT-F	GCTAGGGAGGCCAAGAAGAG	qRT-PCR
CYP703A3-qRT-R	TTGGTCACCGATGATGTGTC	qRT-PCR
CYP704B2-qRT-F	GGCAGAGTTGTAGACATGCA	qRT-PCR
CYP704B2-qRT-R	CGACAGTATGTCGTGCTTGA	qRT-PCR
TDR-qRT-F	GGAGGAGTAACAAGGACCCA	qRT-PCR
TDR-qRT-R	ACCTCCAGCAGCGAGTCCCT	qRT-PCR
TIP2-qRT-F	GTGGAGGACGACGTGAACAT	qRT-PCR
TIP2-qRT-R	CACCACTTCAATCAGCCTGC	qRT-PCR
MTR1-qRT-F	CTCTTTCGTCCTCTGCTTCTAAC	qRT-PCR
MTR1-qRT-R	GGCAGTAGCACGATATGTCATC	qRT-PCR
OsGPAT3-in situ-F	ATCCAACACCAACACCACATTGCC	in situ hybridazation
OsGPAT3-in situ-R	AGAAGGACACCATTGCCATGACCT	in situ hybridazation
OsGPAT3-in situ T7-F	TAATACGACTCACTATAGGG ATCCAACACCAACACCACATTGCC	in situ hybridazation
OsGPAT3-in situ T7-R	TAATACGACTCACTATAGGG AGAAGGACACCATTGCCATGACCT	in situ hybridization
